# Cardiac Biomarkers Predict Major Adverse Cardiac Events (MACE) in Incident Haemodialysis Patients: Results from a Global Federated Database

**DOI:** 10.3390/biomedicines13020367

**Published:** 2025-02-05

**Authors:** Elin Mitford Davies, Benjamin J. R. Buckley, Philip Austin, Gregory Y. H. Lip, Anirudh Rao, Garry McDowell

**Affiliations:** 1 Department of Women’s and Children’s Health, Institute of Life Course and Medical Sciences, University of Liverpool, Liverpool L7 8TX, UK; elin.davies@liverpool.ac.uk; 2 Department of Nephrology, Liverpool University Hospitals NHS Foundation Trust, Liverpool L7 8YE, UK; anirudh.rao@liverpoolft.nhs.uk; 3 Department of Paediatric Nephrology, Alder Hey Children’s NHS Foundation Trust Hospital, Eaton Road, Liverpool L14 5AB, UK; gregory.lip@liverpool.ac.uk; 4 Liverpool Centre for Cardiovascular Science, University of Liverpool, Liverpool John Moores University & Liverpool Heart and Chest Hospital, Liverpool L7 8TX, UK; b.j.buckley@ljmu.ac.uk; 5 Cardiovascular Health Sciences, Research Institute for Sport and Exercise Sciences, Liverpool John Moores University, Liverpool L3 3AF, UK; 6 TriNetX Inc., London EC3V 4AB, UK; philip.austin@trinetx.com; 7 Danish Centre for Health Services Research, Department of Clinical Medicine, Aalborg University, 2450 Aalborg, Denmark; 8 School of Pharmacy and Biomolecular Sciences, Liverpool John Moores University, Liverpool L3 3AF, UK; 9 Institute of Life Course and Medical Sciences, University of Liverpool, Liverpool L7 8TX, UK; 10 Research Laboratory, Liverpool Heart and Chest Hospital, Liverpool L14 3PE, UK

**Keywords:** cardiac biomarkers, troponin, BNP, chronic kidney disease, haemodialysis, prognosis

## Abstract

**Background:** Despite its many advantages, haemodialysis (HD) has been shown to be associated with significant cardiovascular events, especially in patients commencing HD. Currently, there is no specific method to risk-stratify incident HD patients. Blood-based biomarkers provide insight into myocardial injury and stress. We aimed to evaluate the association of increased circulating biomarker concentration in incident HD with incident major adverse cardiac events (MACE). **Methods:** This was a retrospective cohort study of incident haemodialysis cases within 3 months of treatment initiation (≥18 years) from the TriNetX database. Cohorts were grouped by biomarker thresholds: Troponin I: ≥50 ng/L, BNP ≥ 100 pg/mL and 1:1 propensity-score matched for demographic characteristics, baseline cardiovascular risk, laboratory values, and cardiovascular medication. Primary outcome: Incidence of major adverse cardiac events (MACE) censored prior to index event of HD. Secondary outcome: Risk of each individual component of the composite outcome. Cox regression reported hazard ratios (95% CI) for the outcomes. **Results:** In total, 62,206 and 10,476 patients were included in the troponin I and BNP cohorts, respectively. In the troponin I cohort, 5878 developed MACE (HR 1.33 (95% CI 1.26–1.41, *p* < 0.0001)). In the BNP cohort, 1050 developed MACE (HR 1.28 (95% CI 1.13–1.44, *p* < 0.0001)). **Conclusions:** In incident HD, routine clinical laboratory biomarkers can predict incident MACE. The results suggest the clinical need for CV mortality and morbidity risk profiling in incident HD using a combination of clinical and laboratory variables.

## 1. Introduction

Chronic kidney disease (CKD) is ranked the 12th leading cause of death, with cardiovascular disease (CVD) deaths, secondary to CKD, accounting for 4.6% of the total mortality associated with CKD [1,2,3]. The risk of CVD increases alongside the progression of CKD towards stage 5 equating to end-stage kidney disease (ESKD).

Both traditional (hypertension, dyslipidaemia and hyperglycaemia) and non-conventional risk factors (uraemic toxins, pro-inflammatory cytokines) are implicated in the multifactorial mechanisms associated with CVD in CKD [4]. Cardiac muscle remodelling and fibrosis occur with established CKD and its associated pro-inflammatory microenvironment [4]. The incidence of left ventricular hypertrophy (LVH) increases with deteriorating kidney function, with 70–80% of ESKD demonstrating LVH [5].

Despite its many advantages, haemodialysis (HD) is linked to a 10–20-fold excess cardiovascular morbidity and mortality compared to the general population [6,7]. A large observational study showed 48% higher mortality in HD when compared to peritoneal dialysis (PD) within two years of commencing treatment, with peak cardiovascular mortality occurring in the second month post-initiation [8]. The reason for this excess mortality is multifactorial and includes the significant hemodynamic effects of HD, with 20–30% of sessions complicated by significant intradialytic hypotension (IDH) [9]. HD has been shown to reduce coronary blood flow, leading to myocardial ischemia [10]. Recurrent ischemia (myocardial stunning) leads to myocardial functional and structural changes and, eventually, cardiac failure and cardiac arrhythmias [4,11].

Currently, there is no specific method to stratify cardiovascular risk in HD patients, and, therefore, patients are not offered targeted interventions. Circulating blood cardiac biomarkers provide insight into various aspects of cardiovascular structure and function, including myocyte injury, myocyte stress, inflammation and fibrosis. Several conventional cardiac biomarkers, such as troponin (a marker of myocyte injury) and BNP (a marker of myocardial stress), are widely used in the general population; however, their significance in ESKD with HD requires further exploration to support some preliminary reports [12,13,14,15].

The aim of the current study was to evaluate the association of increased circulating biomarker concentration in incident HD with major adverse cardiac events (MACE).

## 2. Materials and Methods

### 2.1. Ethics Statement

This retrospective cohort study was based on de-identified, pseudo-anonymised or limited data from TriNetX (100 Cambridge Park Drive, Suite 501, Cambridge, MA 02140, USA, Access date: 6 April 2024), a global federated health research network, with participating healthcare organisations (HCOs) granting the use of this data for research purposes [16]. Ethical approval for HCO inclusion occurs via an institutional review board or independent ethics committee [16]. TriNetX includes aggregated data that are analysed and reported, and, given that no patient-level data were received, ethical approval was not required for this study.

### 2.2. Data Source, Study Protocol and Patient Selection

The TriNetX data utilised represent the Global Collaborative Network of 127 HCOs of >110 million patients, primarily in North America (52%) and Western Europe (48%). The HCOs encompass hospitals, primary care facilities and specialist clinics and they contain data from both insured and uninsured patients. The real-world data captured within TriNetX allow for global data aggregation, allowing the platform to develop beyond its initial aim to optimise clinical trial design and be used in numerous peer-reviewed publications [16,17]. More information on TriNetX can be found online (https://trinetx.com/, accessed on 6 April 2024).

The dataset included in this study was accessed on 6 April 2024. The data utilised in this study, predominantly derived from electronic health records (EHRs), include demographics, diagnosis (standardised to the International Statistical Classification of Diseases and Related Health Problems 10th Revision, Clinical Modification [ICD-10CM] [18], or the Systematised Nomenclature of Medicine Clinical Terms [SNOMED] codes [19]), laboratory results (Logical Observation Identifiers Names and Codes) [LOINC] [20] and medication (as per the Anatomical Therapeutic Chemical [ATC] classification system [21]). A full list of ICD-10CM, SNOMED and LOINC codes used is shown in Tables S1 and S2.

### 2.3. Study Cohort

All patients with a diagnosis of chronic kidney disease (as coded by ICD-10CM) requiring haemodialysis within 3 months of diagnosis were included.

According to biomarker-specific thresholds, two cohorts were generated for analysis, viz., the following:

1. Troponin I cohorts stratified as troponin I ≥ 50 ng/L or <50 ng/L.
2. B-type natriuretic peptide (BNP) cohorts stratified as BNP ≥ 100 pg/mL or ˂100 pg/mL, respectively.

At the time of the search, 119 HCOs responded, with 59 providers responding with patients in cohort 1 (troponin I) and 52 providers responding with patients in cohort 2 (BNP).

Cardiac biomarkers (troponin I and BNP) were the first reported result within three months of commencing haemodialysis. The specific threshold values chosen reflected data from published research [22,23] on the values of cardiac biomarkers encountered in CKD, thresholds higher than recommended in clinical guidelines for excluding heart failure and acute coronary syndromes [24,25]. It was noted that there is considerable variation in the BNP concentrations in patients with CKD; however, a value of 100 pg/mL has been reported as the minimum threshold [26]. In addition, biomarker thresholds were validated a priori from the experience of 2 authors (AR and GM) using the values encountered in clinical practice.

### 2.4. Index Event

The index event was defined as the commencement of haemodialysis treatment and a cardiac biomarker (troponin I or BNP) measured within 3 months of haemodialysis initiation. The cardiac biomarker result was the first reported result following the commencement of haemodialysis. The index event whereby a patient met the criteria for inclusion could be up to 20 years before the data search date.

### 2.5. Follow-Up and Clinical Outcome

The primary outcome was the incidence of any major adverse cardiac event (MACE) or all-cause mortality (death) by ICD-10CM (Table S3) that occurred between 1 day after the index event and five-year follow-up. A MACE was defined as a composite of ischaemic heart disease (IHD) (ICD-10CM: I20–I25), angina (ICD-10CM: I20), acute myocardial infarction (AMI) (MI ICD-10CM: I21), heart failure (ICD-10CM: I50), atrial fibrillation or flutter (ICD-10CM: I48), ischaemic stroke (ICD-10CM: I63) and all-cause mortality (death). Patients who incurred a MACE prior to the index event were excluded. The secondary outcome was the risk for each component of the composite outcome.

### 2.6. Covariates

To address any disparity within the cohorts, we included demographic data on age, sex, race, smoking status, cardiovascular comorbidity and prescribed cardiovascular medication. Relevant physical examination results that could be associated with the specified outcomes were retrieved including smoking status, Body Mass Index and left ventricular ejection fraction.

Laboratory results were measured as a continuous variable (haemoglobin, serum albumin, alkaline phosphatase, potassium, sodium, calcium, phosphate, cholesterol, low-density lipoprotein cholesterol, high-density lipoprotein-cholesterol and parathyroid hormone).

### 2.7. Statistical Analysis

All statistical analyses were performed on the TriNetX online platform. As a continuous variable, age was expressed as the mean and standard deviation (SD) and tested for differences with an independent-sample t-test. The socio-demographic characteristics, comorbidities, cardiovascular medications and laboratory results were expressed as absolute frequencies and percentages and tested for differences with the chi-squared test.

Before analysis, cohorts were 1:1 propensity score-matched (PSM) [27] for covariates that could potentially confound the primary outcomes; these included the following:

- Socio-demographic characteristics (age at index, gender and race);
- Baseline CVD comorbidities (glomerular diseases (N08), hypertensive diseases (I10–I16), diabetes mellitus (E08–E13), chronic ischaemic heart disease (I25), cardiovascular procedures, smoking status (F17 nicotine dependence) and Body Mass Index (BMI kg/m^2^);
- Laboratory data (haemoglobin, serum albumin, alkaline phosphatase, potassium, sodium, calcium, phosphate, cholesterol, low-density lipoprotein cholesterol, high-density lipoprotein-cholesterol and parathyroid hormone);
- Common cardiovascular medications (β-adrenergic receptor antagonists, antilipaemic agents, angiotensin-converting enzyme inhibitors, angiotensin II receptor antagonists, aspirin and clopidogrel);
- Left ventricular ejection fraction (%).

PSM was performed using the online TriNetX platform. The platform uses ’greedy nearest-neighbour matching’ with a calliper of 0.1 pooled standard deviations and a difference between propensity scores ≤ 0.1. Covariate balance between groups was assessed using standardised mean differences (SMDs) and included in the supplementary results; SMD between cohorts < 0.1 was considered well-matched.

Following PSM, Cox proportional hazard models were used to assess the association between biomarkers above the threshold and the primary and secondary outcomes during 5-year follow-up.

Results were reported as hazard ratios (HRs) with 95% confidence intervals and Kaplan–Meier survival curves with log-rank tests. No imputations were made for missing data due to the restriction of the TriNetX analytical platform. Censoring was applied and a patient was removed (censored) from the analysis after the last event in their electronic record. The TriNetX ‘Analytics’ functionality and the R Survival package v3.2-3 were used for analysis. A *p*-value < 0.05 was accepted as the level of statistical significance.

### 2.8. Exploratory Analysis

We performed 4 additional exploratory analyses to investigate the following:

1. The association of biomarkers and outcomes in patients ≥ 65 years of age.
2. The association of biomarkers and outcomes in patients with BMI ≥ 25 kg/m^2^.
3. The association of biomarkers and outcomes in female patients only.
4. A combined biomarker approach combining both BNP AND troponin I using the thresholds above.

In all cases, the above additional analyses were performed following 1:1 PSM and including the same variables as in the main analysis.

## 3. Results

### 3.1. Demographics

#### 3.1.1. Troponin I

**Figure 1:**
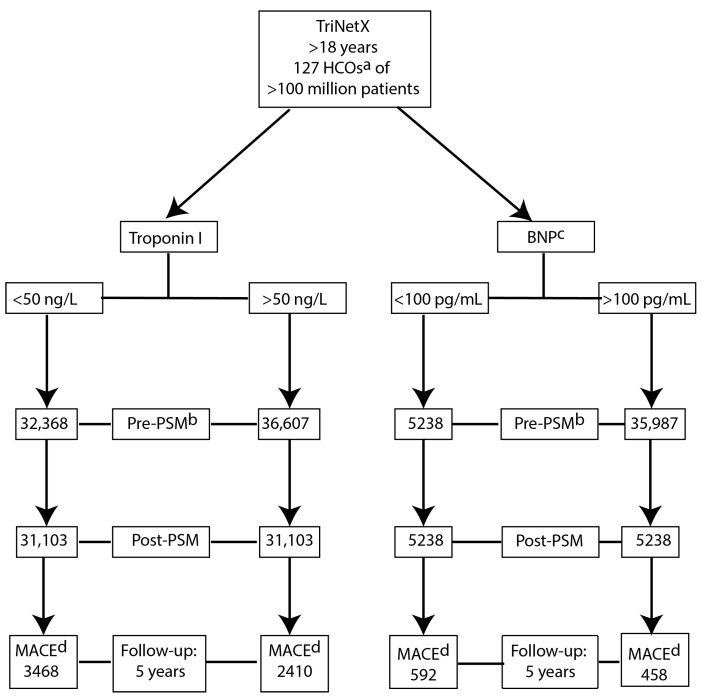
Patient enrolment algorithm. Flowchart demonstrating cohort selection of haemodialysis patients from TriNetX Collaborative Network 6.4.2024. After exclusions, 68,975 haemodialysis patients had troponin I measured; PSM^b^ resulted in 31,103 patients in each group. After exclusions, 41,225 haemodialysis patients had BNP^c^ measured; PSM resulted in 5238 patients in each group. The primary outcome was the incidence of any major adverse cardiac event (MACE) over a five-year follow-up. ^a^ Healthcare organisations, ^b^ PSM—Propensity score-matching, ^c^ B-type natriuretic peptide, ^d^ MACE—Major adverse cardiovascular event.

A total of 68,975 patients meeting our search criteria were identified. Prior to propensity score-matching (PSM), patients with troponin I ≥ 50 ng/L were older; had a higher proportion of males; had a greater prevalence of diabetes mellitus, hypertensive disease and chronic ischaemic heart disease (IHD); and underwent a greater number of cardiovascular procedures. A summary of the PSM characteristics may be found in Table S4a. Following PSM, 62,206 patients were included in the analysis (mean age at index event 61.4 ± 14.2 years; 55% male). The prevalence was 87% for hypertension, 67% for diabetes mellitus and 9.7% for glomerular disorders. Nicotine use was reported in 23.2% of the cohort. Beta-adrenergic receptor antagonists and antilipaemic agents were the most commonly prescribed medications. Following PSM, both cohorts (troponin I ≥ 50 ng/L or <50 ng/L) were well-matched for age, gender and CV risk factors, with no statistically significant differences between groups. Patient selection is illustrated in the study flow diagram (Figure 1).

#### 3.1.2. B-Type Natriuretic Peptide (BNP)

A total of 41,225 patients meeting our search criteria were identified. Prior to propensity score-matching (PSM), patients with BNP I ≥ 100 pg/mL were older, had a higher proportion of males and had a greater prevalence of diabetes mellitus and chronic IHD. A summary of the PSM characteristics may be found in Table S4b. Following PSM, 10,476 patients were included in the analysis (mean age at index event 57.4 ± 14.7 years; 55% male). Prevalence was 90% for hypertension, 67% for diabetes mellitus and 8.7% for glomerular disorders. Nicotine use was reported in 24.8% of the cohort. Beta-adrenergic receptor antagonists and antilipaemic agents were the most commonly prescribed medications. Following PSM, both cohorts (BNP ≥ 100 pg/mL or <100 pg/mL) were well-matched for age, gender and CV risk factors, with no statistically significant differences between groups. Patient selection is illustrated in the study flow diagram (Figure 1).

Patient demographics post propensity score-matching are shown in Table 1a,b.

### 3.2. Clinical Outcomes

#### 3.2.1. Troponin I

**Figure 2:**
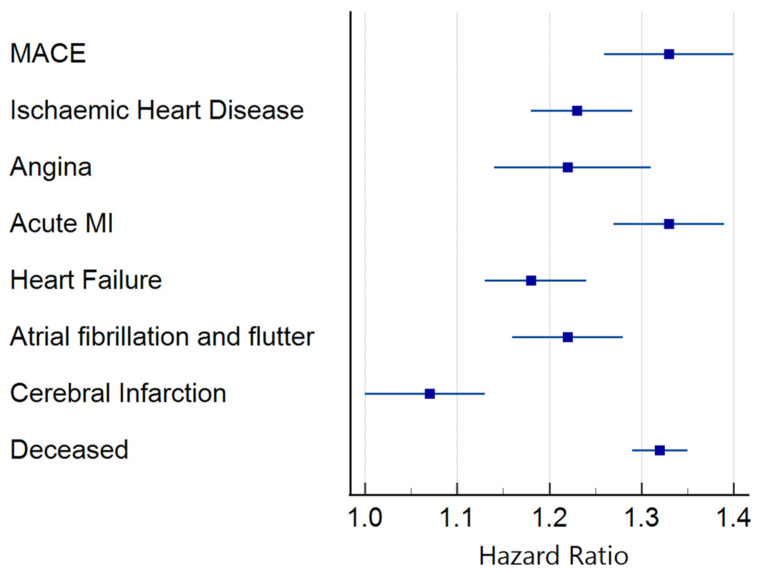
Forrest plot of survival analysis. Hazard ratio and 95% confidence interval for troponin I at a threshold of 50 ng/L.

For the primary outcome of MACE, 9282 of the 62,206 patients had 5-year follow-up data available from the time of the index event. Of these, 5878 developed the primary composite outcome. Of the total number of patients who developed the composite outcome, 2410 had troponin I ≥ 50 ng/L, equating to an HR of 1.33 (1.26–1.40). The results of the secondary outcome analysis showed that increased troponin I was associated with a significantly increased risk of IHD [HR 1.23 (1.18–1.29)], angina pectoris [HR 1.22 (1.14–1.31)], acute myocardial infarction (AMI) [1.33 (1.27–1.39)], heart failure (HF) [HR 1.18 (1.13–1.24)], atrial fibrillation and flutter [HR 1.22 (1.16–1.28)], and all-cause mortality [HR 1.32 (1.29–1.35)] (Figure 2 and Table S5). Only cerebral infarction did not reach the level of statistical significance.

#### 3.2.2. B-Type Natriuretic Peptide (BNP)

**Figure 3:**
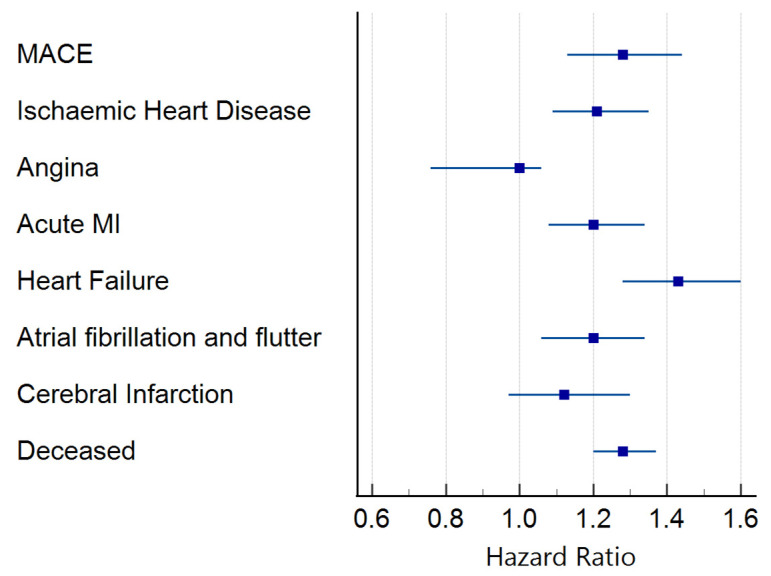
Forrest plot of survival analysis. Hazard ratio and 95% confidence interval for BNP at a threshold of 100 pg/mL.

For the primary outcome of MACE, 1828 of the 10,476 patients had 5-year follow-up data available from the time of the index event. Of these, 1050 developed the primary composite outcome. Of the total number of patients who developed the composite outcome, 458 had BNP ≥100 pg/mL, equating to an HR of 1.28 (1.13–1.44). The results of the secondary outcome analysis showed that increased BNP was associated with a significantly increased risk of IHD [HR 1.21 (1.09–1.35)], AMI [HR 1.20 (1.08–1.34)], HF [HR 1.43 (1.28–1.60)], atrial fibrillation and flutter [HR 1.20 (1.06–1.34)], and all-cause mortality [HR 1.28 (1.20–1.37)] (Figure 3 and Table S6). Only angina pectoris and cerebral infarction did not reach the level of statistical significance.

**Figure 4:**
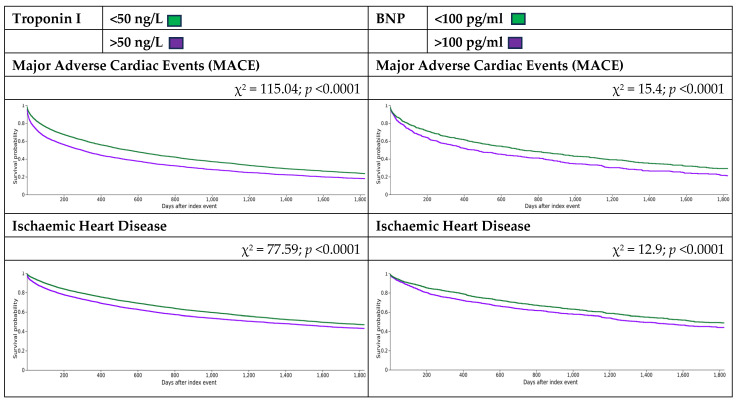

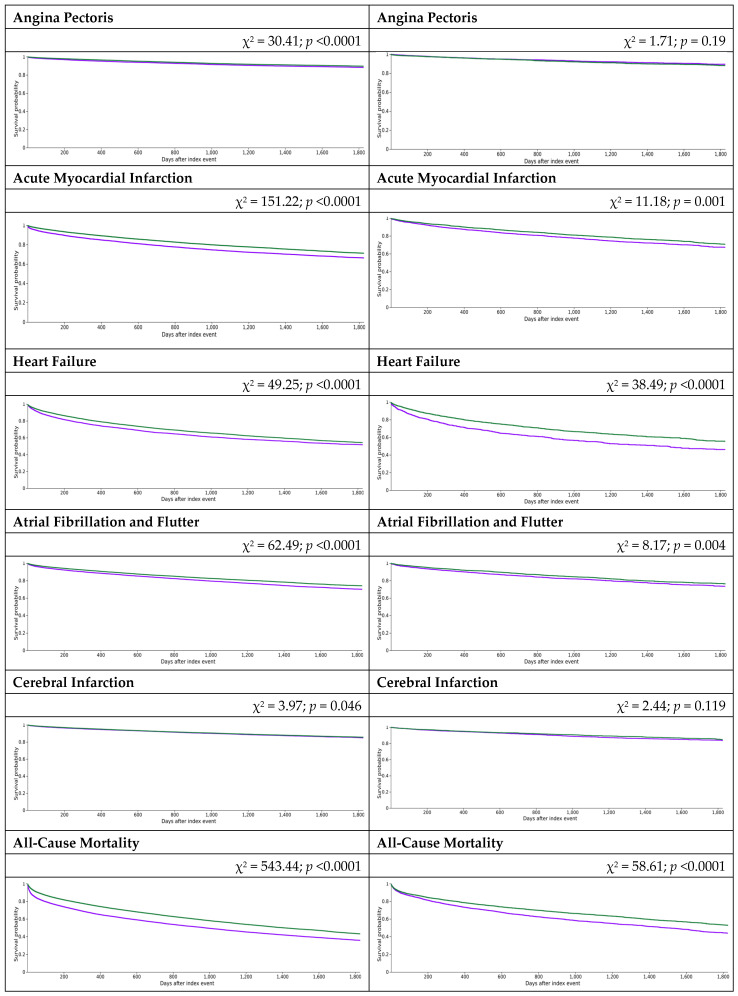
Kaplan–Meier survival analysis curves.

Kaplan–Meier survival analysis (KM) was performed, excluding patients with the outcome prior to the time window, which demonstrated that MACE and their components increased the risk of mortality for incident HD patients, including in the exploratory analysis (Figure 4).

### 3.3. Exploratory Analysis

#### 3.3.1. Troponin I: Patients ≥ 65 Years of Age

For the primary outcome of MACE, 4144 of the 39,132 patients had 5-year follow-up data available from the time of the index event. Of these, 2815 developed the primary composite outcome. Of the total number of patients who developed the composite outcome, 1087 had troponin I ≥ 50 ng/L, equating to an HR of 1.28 (1.19–1.39). The results of the secondary outcome analysis showed that increased troponin I was associated with a significantly increased risk of IHD [HR 1.22 (1.14–1.30)], angina pectoris [HR 1.26 (1.14–1.37)], AMI [HR 1.36 (1.29–1.44)], HF [HR 1.25 (1.18–1.33)], atrial fibrillation and flutter [HR 1.20 (1.13–1.28)], and all-cause mortality [HR 1.32 (1.27–1.35)]. Only cerebral infarction did not reach the level of statistical significance.

#### 3.3.2. B-Type Natriuretic Peptide (BNP): Patients ≥ 65 Years of Age

For the primary outcome of MACE, 617 of the 5132 patients had 5-year follow-up data available from the time of the index event. Of these, 401 developed the primary composite outcome. Of the total number of patients who developed the composite outcome, 175 had BNP ≥ 100 pg/mL, equating to an HR of 1.41 (1.15–1.72). The results of the secondary outcome analysis showed that increased BNP was associated with a significantly increased risk of IHD [HR 1.34 (1.14–1.58)], AMI [HR 1.27 (1.09–1.49)], HF [HR 1.56 (1.32–1.85)], atrial fibrillation and flutter [HR 1.29 (1.10–1.51)], and all-cause mortality [HR 1.35 (1.20–1.48)]. Only angina pectoris and cerebral infarction did not reach the level of statistical significance.

#### 3.3.3. Troponin I: BMI ≥ 25 kg/m^2^

For the primary outcome of MACE, 3433 of the 24,256 patients had 5-year follow-up data available from the time of the index event. Of these, 2075 developed the primary composite outcome. Of the total number of patients who developed the composite outcome, 834 had troponin I ≥ 50 ng/L, equating to an HR of 1.44 (1.32–1.57). The results of the secondary outcome analysis showed that increased troponin I was associated with a significantly increased risk of IHD [HR 1.25 (1.16–1.35)], angina pectoris [HR 1.23 (1.11–1.40)], AMI [HR 1.35 (1.26–1.45)], HF [HR 1.31 (1.22–1.41)], atrial fibrillation and flutter [HR 1.29 (1.19–1.39)], and all-cause mortality [HR 1.43 (1.37–1.49)]. Only cerebral infarction did not reach the level of statistical significance.

#### 3.3.4. B-Type Natriuretic Peptide (BNP): BMI ≥ 25 kg/m^2^

For the primary outcome of MACE, 892 of the 5206 patients had 5-year follow-up data available from the time of the index event. Of these, 521 developed the primary composite outcome. Of the total number of patients who developed the composite outcome, 226 had BNP ≥ 100 pg/mL, equating to an HR of 1.44 (1.20–1.71). The results of the secondary outcome analysis showed that increased BNP was associated with a significantly increased risk of IHD [HR 1.20 (1.04–1.39)], AMI [HR 1.26 (1.09–1.46)], HF [HR 1.41 (1.20–1.65)], atrial fibrillation and flutter [HR 1.29 (1.10–1.52)], and all-cause mortality [HR 1.40 (1.27–1.53)]. Only angina pectoris and cerebral infarction did not reach the level of statistical significance.

#### 3.3.5. Troponin I: Female Only

For the primary outcome of MACE, 3981 of the 27,096 patients had 5-year follow-up data available from the time of the index event. Of these, 2569 developed the primary composite outcome. Of the total number of patients who developed the composite outcome, 1045 had troponin I ≥ 50 ng/L, equating to an HR of 1.32 (1.23–1.43). The results of the secondary outcome analysis showed that increased troponin I was associated with a significantly increased risk of IHD [HR 1.20 (1.08–1.24)], angina pectoris [HR 1.29 (1.16–1.43)], AMI [HR 1.36 (1.28–1.46)], cerebral infarction [HR 1.12 (1.03–1.22)], HF [HR 1.21 (1.13–1.30)], atrial fibrillation and flutter [HR 1.23 (1.14–1.32)], and all-cause mortality [HR 1.36 (1.31–1.41)].

#### 3.3.6. B-Type Natriuretic Peptide (BNP): Female Only

For the primary outcome of MACE, 867 of the 4778 patients had 5-year follow-up data available from the time of the index event. Of these, 533 developed the primary composite outcome. Of the total number of patients who developed the composite outcome, 245 had BNP ≥ 100 pg/mL, equating to an HR of 1.35 (1.14–1.61). The results of the secondary outcome analysis showed that increased BNP was associated with a significantly increased risk of IHD [HR 1.19 (1.03–1.38)], AMI [HR 1.33 (1.14–1.55)], HF [HR 1.45 (1.24–1.71)], atrial fibrillation and flutter [HR 1.25 (1.06–1.49)], and all-cause mortality [HR 1.36 (1.26–1.52)]. Only angina pectoris and cerebral infarction did not reach the level of statistical significance.

#### 3.3.7. Combined Biomarkers (Troponin I and BNP)

For the primary outcome of MACE, 707 of 5386 patients had 5-year follow-up data available from the time of the index event. Of these, 469 developed the primary composite outcome. Of the total number of patients who developed the composite outcome, 194 had troponin I ≥ 50 ng/L AND BNP > 100 pg/mL, equating to an HR of 1.64 (1.38–1.98). The results of the secondary outcome analysis showed that increased troponin I AND BNP was associated with a significantly increased risk of IHD [HR 1.46 (1.26–1.69)], AMI [HR 1.53 (1.34–1.76)], HF [HR 1.66 (1.39–1.88)], atrial fibrillation and flutter [HR 1.37 (1.19–1.60)], and all-cause mortality [HR 1.52 (1.4–1.66)]. Only angina pectoris and cerebral infarction did not reach the level of statistical significance.

## 4. Discussion

This study reports a large retrospective cohort of the prognostic significance of routinely measured cardiac biomarkers. This study was based on a large, multi-million patient database from participating healthcare organisations across four continents. As such, this study is reflective of clinical practice and is generalisable globally. The biomarkers evaluated are already used in clinical practice and can be measured easily in hospital diagnostic laboratories.

The results of the current, large, multicentre cohort study indicate that routine clinical laboratory cardiac biomarkers, BNP or cardiac troponin I, can predict incident MACE in incident haemodialysis, including in patients ≥65 years of age, with BMI ≥25 and in a female-only cohort. In patients commencing HD, increased BNP or cardiac troponin I produced a statistically significant correlation with incident MACE, which complements and expands current research in the prevalent dialysis population.

In this study, we focused the analysis on routinely available laboratory biomarkers, including BNP and troponin I. Routine circulating plasma biomarkers provide insight into various aspects of cardiovascular function, including myocyte injury (troponin) and myocyte stress (BNP).

Cardiac troponin is a recognised biomarker of myocardial injury and is incorporated in international guidelines for AMI [25]. Previous work from one of the authors (GM) demonstrated that increased cardiac troponin in pre-dialysis end-stage kidney disease patients was predictive of 2-year survival [28]. This work has been expanded by other authors in the HD population, who have reported the prognostic significance of cardiac troponin, measured by conventional and high-sensitivity assays, although these studies are limited by relatively small sample sizes [29,30,31,32,33], and, additionally, the majority of these were conducted in prevalent dialysis populations. Our study reported here investigated the incident HD population and reported an HR of 1.33 [(1.26–1.40); X^2^ 115.04; *p* < 0.0001] for MACE and 1.32 [(1.29–1.35); X^2^ 543.43; *p* < 0.0001] for all-cause mortality. Our results are consistent with outcomes reported by Shafi et al. [34] in their prospective study of a smaller cohort. Our study has the further advantage of being representative of real-life clinical practice.

Previous studies have postulated the mechanism of increased cardiac troponin in HD patients, many of which have now been disproven or otherwise considered unlikely [35]. The mechanism of increased cardiac troponin in this population remains to be fully understood, is most likely multifactorial (28) and may result from myocyte injury/necrosis. Additionally, recent work has demonstrated a correlation between circulating plasma levels of troponin T and echocardiographic left ventricular dysfunction [33,36], and it is well-recognised that patients with ESKD are more likely to develop HF [37]. Haemodialysis is associated with myocardial stunning, which has been associated with mortality. Similarly, viable myocytes have been shown to release troponin [38,39]. Irrespective of the mechanism, increased circulating plasma levels of troponin T and I have been shown to be predictive of an increased risk of all-cause mortality and MACE [11,40] (other mechanisms include myocardial stunning, LVH, myocardial micronecrosis and cell membrane permeability changes [41,42,43]).

The natriuretic peptides are similarly incorporated into international guidelines for HF [24] and have been shown to predict outcomes in patients with established HF [44]. Circulating plasma BNP and its N-terminal precursor (NTproBNP) are released by ventricular myocytes in response to myocyte stress and increased transmural pressure [45]. Previous studies have demonstrated the prognostic significance of an increased circulating plasma concentration of BNP and its N-terminal precursor in haemodialysis, again, mainly in the prevalent HD population, with all studies being limited by a relatively small sample size [30,31,32,33,34,46,47,48,49]. In our current study of incident HD, BNP concentrations ≥ 100 pg/mL were associated with an HR for MACE of 1.28 [(1.13–1.44); X^2^ 15.36; *p* < 0.0001] and an HR for all-cause mortality of 1.28 [(1.20–1.37); X^2^ 58.61; *p* < 0.0001], which is consistent with results reported for the prevalent HD population.

Similar to troponin, the reason for increased BNP in HD is most likely multifactorial and possibly related to volume overload and LV dysfunction, which is common in HD patients [46].

In addition, considerable evidence demonstrates the effect of age, sex and BMI on circulating plasma levels of cardiac biomarkers [50,51]. There is a weak inverse correlation between BMI and NTproBNP, although current guidelines support the use of NTproBNP in the diagnosis of HF regardless of BMI [52,53]. Due to this, we decided to perform three additional exploratory analyses evaluating age, BMI and gender. While there were differences between groups, troponin I and BNP remained predictive of the risk of MACE and all-cause mortality.

Studies of the predictive utility of routine cardiac biomarkers on CV outcomes have utilised a single-sample strategy. While this is compelling, it does not take into account the normal biological variation in circulating plasma concentrations, nor the analytical variation associated with their measurement. In a study of 171 patients with stable HD, Chesnaye et al. [54] reported that patients with troponin T that remained elevated or fluctuated were associated with a worse cardiac outcome. In a rather unique approach, Sandoval and co-authors [55] explored reference change values (RCVs) to control for biological and analytical variation and reported that an increase in cardiac troponin over a 3-month period greater than the RCV was predictive of an increased risk of all-cause mortality in HD patients.

### Strengths and Limitations

The study methodology was governed by the available statistical analysis adopted by TriNetX. The platform does not allow weighting to be applied to specific covariates, and the final selected cohort was restricted only to those who had a complete set of co-variables. Consequently, there is a risk of overestimation.

Dependence on disease classification codes is a limitation that cannot be overcome and can occasionally lead to misclassification. However, all data were semantically harmonised to TriNetX interface terminology, which utilises predominantly ICD-10 codes [16].

While real-world data reflect clinical practice, the retrospective nature of this study means that the cohorts were not randomised or controlled. However, using a quasi-experimental approach with PSM replicates a randomised control trial within observational data, somewhat mitigating the risk [56]. The data were derived from electronic health records for administrative purposes; therefore, there was the potential for data errors or missing data. Patients/data may also be lost at follow-up if a patient moves to a different healthcare organisation, which could potentially skew covariate distribution and outcomes.

PSM balanced cohorts for age, gender and CV risk factors. However, omitting socio-economic data such as deprivation indices and family history could have biased the results.

In considering the context of our work, it is important to consider the biomarker thresholds chosen. In our analysis, our thresholds were chosen based on the experience of two authors (AR and GM) and validated by reference to the published literature. Previous studies have utilised a threshold value or, as appears for the majority, used the biomarker as a continuous variable. Of particular note, the efficiency of dialysis or patients’ fluid status was not included in the analysis, which may also have affected the results, particularly for BNP [57]. However, NTproBNP has been shown to be independent of fluid status [58]. In addition, it was not possible to identify the timing of the samples included and ascertain if these were pre- or post-dialysis.

In addition, over the time frame of the current study, the analytical sensitivity of the cardiac troponin I assay has improved. The improved analytical sensitivity of more recent troponin I assays, however, should not have impacted the threshold values chosen as these were within the analytical range of so-called conventional and high-sensitivity cardiac troponin assays. Further, and in addition, we repeated the analysis with high-sensitivity troponin I assays alone. The results of these supported the validity of our initial analysis. For the primary outcome of MACE, 2158 of 9208 patients had 5-year follow-up data available from the time of the index event. Of these,1515 developed the primary composite outcome. Of the total number of patients who developed the composite outcome, 717 had hs-troponin I ≥ 50 ng/L, equating to an HR of 1.15 (1.04–1.27). The results of the secondary outcome analysis showed that increased hs-troponin I was associated with a significantly increased risk of IHD [HR 1.25 (1.14–1.37)], AMI [HR 1.68 (1.53–1.84)], HF [HR 1.28 (1.17–1.41)], atrial fibrillation and flutter [HR 1.29 (1.16–1.44)], and all-cause mortality [HR 1.36 (1.26–1.57)]. Only angina pectoris and cerebral infarction did not reach the level of statistical significance.

## 5. Conclusions

Routinely available cardiac biomarkers can predict incident MACE in incident HD. A combined biomarker approach also adds additional risk prediction. The results of this large, retrospective cohort study suggest the clinical need for CV mortality and risk profiling in patients commencing HD using a combination of clinical and laboratory variables.

## Figures and Tables

**Table 1:** (a) Patient demographics and cardiovascular risk factor profile post-propensity score-matching stratified by cardiac troponin I at a threshold of 50 ng/L. (b) Patient demographics and cardiovascular risk factor profile post-propensity score-matching stratified by BNP at a threshold of 100 pg/mL.

(a)				
	Troponin I			
	≥50 ng/L	<50 ng/L	*p*-Value	Std Diff.
**Sample size**	31,103	31,103		
**Demographics**				
Age at index Mean ± SD	61.4 ± 14.1	61.3 ± 14.2	0.453	0.006
Male N (%)	17,034 (54.8%)	17,103 (55.0%)	0.578	0.004
White N (%)	13,722 (44.1%)	13,561 43.6%	0.193	0.010
American Indian or Alaska Native N (%)	156 (0.5%)	170 (0.5%)	0.437	0.006
Native Hawaiian or other Pacific Islander N (%)	501 (1.6%)	548 (1.8%)	0.143	0.012
Black or African American N (%)	12,167 (39.1%)	12,136 (39.0%)	0.799	0.002
Asian N (%)	1459 (4.7%)	1477 (4.7%)	0.734	0.003
Other race N (%)	1052 (3.4%)	1082 (3.5%)	0.509	0.005
**Co-morbidities**				
Diabetes mellitus N (%)	21,002 (67.5%)	20,901 (67.2%)	0.388	0.007
Glomerular disorders N (%)	3076 (9.9%)	2924 (9.4%)	0.039	0.017
Hypertensive diseases N (%)	27,136 (87.2%)	27,067 (87.0%)	0.409	0.007
Smoker N (%)	7282 (23.4%)	7178 (23.1%)	0.324	0.008
Chronic ischemic heart disease N (%)	17,251 (55.5%)	15,448 (49.7%)	<0.001	0.116
Cardiovascular procedures N (%)	27,295 (87.8%)	26,835 (86.3%)	<0.001	0.044
**Medications**				
Beta-adrenergic receptor antagonists N (%)	24,448 (78.6%)	24,908 (80.1%)	<0.001	0.037
Antilipaemic agents N (%)	19,711 (63.4%)	19,704 (63.4%)	0.954	<0.001
ACE inhibitors N (%)	12,995 (41.8%)	13,268 (42.7%)	0.027	0.018
Angiotensin II receptor antagonists N (%)	8957 (28.8%)	9291 (29.9%)	0.003	0.024
Aspirin N (%)	19,799 (63.7%)	19,259 (61.9%)	<0.001	0.036
Clopidogrel N (%)	7738 (24.9%)	6970 (22.4%)	<0.001	0.058
**Laboratory Results**				
Haemoglobin (g/dL)	9.7 ± 1.9	9.6 ± 1.8	0.094	0.014
Albumin (g/dL)	3.2 ± 0.7	3.2 ± 0.7	0.806	0.002
Alkaline phosphatase (U/L)	127.5 ± 121.6	124.5 ± 110.5	0.002	0.026
Potassium (mmol/L)	4.4 ± 0.7	4.4 ± 0.7	0.001	0.026
Sodium (mmol/L)	136.4 ± 4.1	136.5 ± 3.9	0.001	0.027
Calcium (mg/dL)	8.6 ± 0.9	8.6 ± 0.9	0.174	0.011
Phosphate (mg/dL)	4.8 ± 1.8	4.6 ± 1.7	<0.001	0.107
Cholesterol (mg/dL)	149.3 ± 55.7	150.8 ± 55.8	0.009	0.027
HDL-C (mg/dl)	42.1 ± 17.3	42.6 ± 17.5	0.007	0.028
LDL-C (mg/dL)	80.1 ± 42.7	80.5 ± 43.2	0.427	0.008
PTH (pg/mL)	347.5 ± 371.76	334.6 ± 353.6	0.002	0.036
BMI (kg/m^2^)	28.7 ± 7.1	28.8 ± 7.2	0.546	0.008
LVEF (%)	51.1 ± 15.3	54.3 ± 14.0	<0.001	0.217
**(b)**				
	**BNP**			
	**≥100 pg/mL**	**<100 pg/mL**	***p*-Value**	**Std diff.**
**Sample size**	**5238**	**5238**		
**Demographics**				
Age at index Mean ± SD	57.5 ± 14.5	57.2 ± 14.8	0.341	0.019
Male N (%)	2853 (54.5%)	2854 (54.5%)	0.984	<0.001
White N (%)	1943 (37.1%)	1935 (36.9%)	0.871	0.003
American Indian or Alaska Native N (%)	11 (0.2%)	15 (0.3%)	0.432	0.015
Native Hawaiian or other Pacific Islander N (%)	73 (1.4%)	77 (1.5%)	0.742	0.006
Black or African American N (%)	2700 (51.5%)	2674 (51.1%)	0.611	0.010
Asian N (%)	164 (3.1%)	170 (3.2%)	0.739	0.007
Other race N (%)	105 (2.0%)	112 (2.1%)	0.631	0.009
**Co-morbidities**				
Diabetes mellitus N (%)	3551 (67.8%)	3489 (66.6%)	0.197	0.025
Glomerular disorders N (%)	446 (8.5%)	466 (8.9%)	0.488	0.014
Hypertensive diseases N (%)	4748 (90.6%)	4693 (89.6%)	0.072	0.035
Smoker N (%)	1301 (24.8%)	1301 (24.8%)	1	<0.001
Chronic ischemic heart disease N (%)	2622 (50.1%)	2347 (44.8%)	<0.001	0.105
Cardiovascular procedures N (%)	4716 (90.0%)	4700 (89.7%)	0.604	0.010
**Medications**				
Beta-adrenergic receptor antagonists N (%)	4380 (83.6%)	4281 (81.7%)	0.011	0.050
Antilipaemic agents N (%)	3294 (62.9%)	3282 (62.7%)	0.808	0.005
ACE inhibitors N (%)	2242 (42.8%)	2218 (42.3%)	0.635	0.009
Angiotensin II receptor antagonists N (%)	1611 (30.8%)	1653 (31.6%)	0.376	0.017
Aspirin N (%)	3344 (63.8%)	3274 (62.5%)	0.156	0.028
Clopidogrel N (%)	1142 (21.8%)	1052 (20.1%)	0.031	0.042
**Laboratory Results**				
Haemoglobin (g/dL)	9.5 ± 1.8	9.9 ± 1.9	<0.001	0.242
Albumin (g/dL)	3.1 ± 0.7	3.2 ± 0.7	<0.001	0.150
Alkaline phosphatase (U/L)	129.0 ± 126.9	120.2 ± 101.3	<0.001	0.077
Potassium (mmol/L)	4.4 ± 0.7	4.4 ± 0.7	0.474	0.014
Sodium (mmol/L)	136.7 ± 4.0	136.8 ± 3.9	0.034	0.042
Calcium (mg/dL)	8.7 ± 0.9	8.8 ± 0.9	<0.001	0.118
Phosphate (mg/dL)	4.7 ± 1.8	4.7 ± 1.8	0.144	0.030
Cholesterol (mg/dL)	150.0 ± 55.3	152.6 ± 55.4	0.050	0.047
HDL-C (mg/dl)	42.8 ± 17.7	41.8 ± 17.2	0.026	0.053
LDL-C (mg/dL)	80.9 ± 44.7	81.4 ± 44.0	0.663	0.010
PTH (pg/mL)	367.4 ± 391.8	341.6 ± 360.6	0.011	0.069
BMI (kg/m^2^)	29.1 ± 7.4	30.1 ± 7.9	<0.001	0.130
LVEF (%)	51.6 ± 15.5	56.6 ± 13.6	<0.001	0.339

## Data Availability

The original contributions presented in this study are included in the article/supplementary material. Further inquiries can be directed to the corresponding author.

## Supplementary Materials

The following supporting information can be downloaded at https://www.mdpi.com/article/10.3390/biomedicines13020367/s1: Table S1: Incident dialysis cohort; Table S2: Procedure codes; Table S3: Clinical outcome codes; Table S4a: Patient demographics and cardiovascular risk factor profile post-propensity score-matching stratified by cardiac troponin I at a threshold of 50 ng/L; Table S4b: Patient demographics and cardiovascular risk factor profile post-propensity score-matching stratified by BNP at a threshold of 100 pg/mL; Table S5: Survival analysis. Number of patients with each MACE outcome, hazard ratio and 95% confidence interval for troponin I at a threshold of 50 ng/L; Table S6: Survival analysis. Number of patients with each MACE outcome, hazard ratio and 95% confidence interval for BNP at a threshold of 100 pg/mL.
