# Supplementary material for: Cardiac Biomarkers Predict Major Adverse Cardiac Events (MACE) in Incident Haemodialysis Patients: Results from a Global Federated Database
Source: Biomedicines. 2025 Feb 5;13(2):367. doi: 10.3390/biomedicines13020367 (PMC11853129; doi:10.3390/biomedicines13020367)
Supplement: Supplementary file 1 [file biomedicines-13-00367-s001.zip › biomedicines-3415609-supplementary.pdf]

Supplementary File

Table S1: Incident Dialysis Cohort

ICD-10 Clinical Modification Code	Description
N18.5	Chronic kidney disease, stage 5
N18.3	Chronic kidney disease, stage 3 (moderate)
N18.32	Chronic kidney disease, stage 3b
N18.4	Chronic kidney disease, stage 4 (severe)
N18.30	Chronic kidney disease, stage 3 unspecified
N18.31	Chronic kidney disease, stage 3a
N18.6	End stage renal disease

Table S2: Procedure Codes

Code	Procedure
CPT:1006747	Hemodialysis Access, Intervascular Cannulation for Extracorporeal Circulation, or Shunt Insertion Procedures on Arteries and Veins
CPT:90940	Hemodialysis access flow study to determine blood flow in grafts and arteriovenous fistulae by an indicator method
SNOMED:302497006	Hemodialysis
CPT:90937	Hemodialysis procedure requiring repeated evaluation(s) with or without substantial revision of dialysis prescription
ICD9CM:39.95	Hemodialysis
CPT:1012752	Hemodialysis Procedures
CPT:90935	Hemodialysis procedure with single evaluation by a physician or other qualified health care professional
CPT:1012752	Hemodialysis Procedures
**Clinical Laboratory Codes**	
TNX:9003	Natriuretic peptide B [Mass/volume] in Serum, Plasma or Blood
LNC:89579-7	Troponin I. cardiac [Mass/volume] in Serum or Plasma by High sensitivity method
TNX:9005	Troponin I. cardiac [Mass/volume] in Serum, Plasma or Blood
TNX: 9014	Haemoglobin
TNX: 9045	Albumin
TNX: 9046	Alkaline phosphatase
TNX: 9028	Potassium
TNX: 9029	Sodium
TNX: 9022	Calcium
TNX: 9027	Phosphate
TNX: 9000	Cholesterol (Total)
TNX: 9001	Cholesterol in HDL
TNX: 9002	Cholesterol in LDL
TNX: 9039	PTH
TNX: 2003	Left Ventricular Ejection Fraction (LVEF)
TNX: 9083	BMI
**Medications**	
CV100	Beta-blocker related
CV350	Antilipaemic agents
CV800	ACE Inhibitors
CV805	Angiotensin II Inhibitors
1191	Aspirin
32968	Clopidogrel

Table S3: Clinical Outcome codes

ICD-10 Clinical Modification Code	Description
ICD10CM:I20-I25	Ischemic heart diseases
ICD10CM:I20	Angina pectoris
ICD10CM:I21	Acute myocardial infarction
ICD10CM:I50	Heart failure
ICD10CM:I48	Atrial fibrillation and flutter
ICD10CM:I63	Cerebral infarction
Deceased	Deceased
Major Adverse Cardiac Event	I20-25 OR I20 OR I21 OR I50 OR I48 OR I63 OR Deceased

Table S4a. Patient demographics and cardiovascular risk factor profile post propensity score matching stratified by cardiac troponin I at a threshold of 50ng/L.

	Troponin I<br>Initial Population (Pre-PSM)				Troponin I<br>Propensity Score Matched (PSM) Population			
	≥50g/L	<50 ng/L	P-Value	Std diff.	≥50 ng/L	<50 ng/L	P-Value	Std diff.
**Demographics**								
**Sample Size**	36,607	32,368			31,103	31,103		
**Age at Index Mean ± SD**	62.7 ± 14.1	60.6 ± 14.5	<0.001	0.143	61.4 ± 14.1	61.3 ± 14.2	0.453	0.006
**Male N (%)**	21,334 (58.3%)	17,360 (53.6%)	<0.001	0.094	17,034 (54.8%)	17,103 (55.0%)	0.578	0.004
**White N (%)**	15,792 (43.1%)	14,067 (43.5%)	0.397	0.006	13,722 (44.1%)	13,561 43.6%	0.193	0.010
**American Indian or Alaska Native N (%)**	185 (0.5%)	180 (0.6%)	0.359	0.007	156 (0.5%)	170 (0.5%)	0.437	0.006
**Native Hawaiian or Other Pacific Islander N (%)**	945 (2.6%)	548 (1.7%)	<0.001	0.061	501 (1.6%)	548 (1.8%)	0.143	0.012
**Black or African American N (%)**	14,016 (38.3%)	12,548 (38.8%)	0.197	0.010	12,167 (39.1%)	12,136 (39.0%)	0.799	0.002
**Asian N (%)**	2,193 (6.0%)	1,483 (4.6%)	<0.001	0.063	1,459 (4.7%)	1,477 (4.7%)	0.734	0.003
**Other Race N (%)**	1,101 (3.0%)	1,281 (4.0%)	<0.001	0.052	1,052 (3.4%)	1,082 (3.5%)	0.509	0.005
**Co-morbidities**								
**Diabetes mellitus N (%)**	24,290 (66.4%)	21,773 (67.3%)	0.011	0.019	21,002 (67.5%)	20,901 (67.2%)	0.388	0.007

**Glomerular disorders N (%)**	3,431 (9.4%)	3,089 (9.5%)	0.444	0.006	3,076 (9.9%)	2,924 (9.4%)	0.039	0.017
**Hypertensive diseases N (%)**	31,712 (86.6%)	28,220 (87.2%)	0.031	0.017	27,136 (87.2%)	27,067 (87.0%)	0.409	0.007
**Smoker N (%)**	8,077 (22.1%)	7,587 (23.4%)	<0.001	0.033	7,282 (23.4%)	7,178 (23.1%)	0.324	0.008
**Chronic ischemic heart disease N (%)**	20,509 (56.0%)	15,862 (49.0%)	<0.001	0.141	17,251 (55.5%)	15,448 (49.7%)	<0.001	0.116
**Cardiovascular Procedures N (%)**	31,936 (87.2%)	27,920 (86.3%)	<0.001	0.029	27,295 (87.8%)	26,835 (86.3%)	<0.001	0.044
**Medications**								
**Beta-adrenergic receptor antagonists N (%)**	28,530 (77.9%)	25,948 (80.2%)	<0.001	0.055	24,448 (78.6%)	24,908 (80.1%)	<0.001	0.037
**Antilipaemic agents N (%)**	23,176 (63.3%)	20,358 (62.9%)	0.260	0.009	19,711 (63.4%)	19,704 (63.4%)	0.954	<0.001
**ACE Inhibitors N (%)**	15,044 (41.1%)	13,879 (42.9%)	<0.001	0.036	12,995 (41.8%)	13,268 (42.7%)	0.027	0.018
**Angiotensin II Receptor Antagonists N (%)**	10,495 (28.7%)	9,663 (29.9%)	0.001	0.026	8,957 (28.8%)	9,291 (29.9%)	0.003	0.024
**Aspirin N (%)**	23,233 19,917	(63.5%) (61.5%)	<0.001	0.040	19,799 (63.7%)	19,259 (61.9%)	<0.001	0.036
**Clopidogrel N (%)**	9,131 (24.9%)	7,147 (22.1%)	<0.001	0.068	7,738 (24.9%)	6,970 (22.4%)	<0.001	0.058

**Laboratory Results**								
**Haemoglobin (g/dL)**	9.7 ± 1.9	9.6 ± 1.8	0.001	0.025	9.7 ± 1.9	9.6 ± 1.8	0.094	0.014
**Albumin (g/dL)**	3.2 ± 0.7	3.2 ± 0.7	0.206	0.010	3.2 ± 0.7	3.2 ± 0.7	0.806	0.002
**Alkaline Phosphatase (U/L)**	125.9 ± 118.8	125.3 ± 111.9	0.569	0.005	127.5 ± 121.6	124.5 ± 110.5	0.002	0.026
**Potassium (mmol/L)**	4.4 ± 0.7	4.4 ± 0.7	0.006	0.022	4.4 ± 0.7	4.4 ± 0.7	0.001	0.026
**Sodium (mmol/L)**	136.4 ± 4.1	136.5 ± 3.9	0.025	0.018	136.4 ± 4.1	136.5 ± 3.9	0.001	0.027
**Calcium (mg/dL)**	8.6 ± 0.9	8.6 ± 0.9	0.051	0.016	8.6 ± 0.9	8.6 ± 0.9	0.174	0.011
**Phosphate (mg/dL)**	4.8 ± 1.8	4.6 ± 1.7	<0.001	0.084	4.8 ± 1.8	4.6 ± 1.7	<0.001	0.107
**Cholesterol (mg/dL)**	148.2 ± 54.9	151.4 ± 56.0	<0.001	0.058	149.3 ± - 55.7	150.8 ± 55.8	0.009	0.027
**HDL-C (mg/dL)**	42.0 ± 17.2	42.7 ± 17.5	<0.001	0.040	42.1 ± 17.3	42.6 ± 17.5	0.007	0.028
**LDL-C (mg/dL)**	79.4 ± 42.1	80.9 ± 43.3	0.001	0.033	80.1 ± 42.7	80.5 ± 43.2	0.427	0.008
**PTH (pg/mL)**	341.3 ± 363.9	335.7 ± 355.1	0.154	0.016	347.5 ± 371.7 6	334.6 ± 353.	0.002	0.036
Clinical Measurements								
**BMI (kg/m²)**	28.6 ± 7.0	28.8 ± 7.2	0.040	0.025	28.7 ± 7.1	28.8 ± 7.2	0.546	0.008
**LVEF (%)**	50.9 ± 15.5	54.3 ± 14.0	<0.001	0.232	51.1 ± 15.3	54.3 ± 14.0	<0.001	0.217

Table S4b. Patient demographics and cardiovascular risk factor profile post propensity score matching stratified by BNP at a threshold of 100 pg/mL.

	BNP				BNP			
	Initial Population (Pre-PSM)				Propensity Score Matched (PSM) Population			
	≥100 pg/mL	<100 pg/mL	P-Value	Std diff.	≥100 pg/mL	<100 pg/mL	P-Value	Std diff.
**Demographics**								
**Sample Size**	35,987	5,238			**5238**	**5238**		
**Age at Index Mean ± SD**	62.2 ± 14.5	57.2 ± 14.8	<0.001	0.340	57.5 ± 14.5	57.2 ± 14.8	0.341	0.019
**Male N (%)**	19,665 (54.6%)	2,854 (54.5%)	0.830	0.003	2,853 (54.5%)	2,854 (54.5%)	0.984	<0.001
**White N (%)**	16,734 (46.5%)	1,935 (36.9%)	<0.001	0.195	1,943 (37.1%)	1,935 (36.9%)	0.871	0.003
**American Indian or Alaska Native N (%)**	121 (0.3%)	15 (0.3%)	0.557	0.009	11 (0.2%)	15 (0.3%)	0.432	0.015
**Native Hawaiian or Other Pacific Islander N (%)**	875 (2.4%)	77 (1.5%)	<0.001	0.070	73 (1.4%)	77 (1.5%)	0.742	0.006
**Black or African American N (%)**	12,760 (35.5%)	2,674 (51.1%)	<0.001	0.319	2,700 (51.5%)	2,674 (51.1%)	0.611	0.010
**Asian N (%)**	2,121 (5.9%)	170 (3.2%)	<0.001	0.127	164 (3.1%)	170 (3.2%)	0.739	0.007
**Other Race N (%)**	952 (2.6%)	112 (2.1%)	0.031	0.033	105 (2.0%)	112 (2.1%)	0.631	0.009
**Co-morbidities**								
**Diabetes mellitus N (%)**	24,663 (68.5%)	3,489 (66.6%)	0.005	0.041	3,551 (67.8%)	3,489 (66.6%)	0.197	0.025

**Glomerular disorders N (%)**	3,002 (8.3%)	466 (8.9%)	0.177	0.020	446 (8.5%)	466 (8.9%)	0.488	0.014
**Hypertensive diseases N (%)**	31,791 (88.3%)	4,693 (89.6%)	0.008	0.040	4,748 (90.6%)	4,693 (89.6%)	0.072	0.035
**Smoker N (%)**	7,964 (22.1%)	1,301 (24.8%)	<0.001	0.064	1,301 (24.8%)	1,301 (24.8%)	1	<0.001
**Chronic ischemic heart disease N (%)**	19,369 (53.8%)	2,347 (44.8%)	<0.001	0.181	2,622 (50.1%)	2,347 (44.8%)	<0.001	0.105
**Cardiovascular Procedures N (%)**	31,841 (88.5%)	4,700 (89.7%)	0.008	0.040	4,716 (90.0%)	4,700 (89.7%)	0.604	0.010
**Medications**								
**Beta-adrenergic receptor antagonists N (%)**	29,409 (81.7%)	4,281 (81.7%)	0.988	<0.001	4,380 (83.6%)	4,281 (81.7%)	0.011	0.050
**Antilipaemic agents N (%)**	23,470 (65.2%)	3,282 (62.7%)	<0.001	0.053	3,294 (62.9%)	3,282 (62.7%)	0.808	0.005
**ACE Inhibitors N (%)**	14,915 (41.4%)	2,218 (42.3%)	0.217	0.018	2,242 (42.8%)	2,218 (42.3%)	0.635	0.009
**Angiotensin II Receptor Antagonists N (%)**	11,205 (31.1%)	1,653 (31.6%)	0.538	0.009	1,611 (30.8%)	1,653 (31.6%)	0.376	0.017
**Aspirin N (%)**	23,064 (64.1%)	3,274 (62.5%)	0.026	0.033	3,344 (63.8%)	3,274 (62.5%)	0.156	0.028
**Clopidogrel N (%)**	8,743 (24.3%)	1,052 (20.1%)	<0.001	0.101	1,142 (21.8%)	1,052 (20.1%)	0.031	0.042
**Laboratory Results**								

**Haemoglobin (g/dL)**	9.6 ± 1.8	9.9 ±1.9	<0.001	0.202	9.5 ± 1.8	9.9 +/- 1.9	<0.001	0.242
**Albumin (g/dL)**	3.1 ± 0.7	3.2 ± 0.7	<0.001	0.165	3.1 ± 0.7	3.2 +/- 0.7	<0.001	0.150
**Alkaline Phosphatase (U/L)**	124.5 ± 120.2	120.2 ± 101.3	0.019	0.038	129.0 ±- 126.9	120.2 +/- 101.3	<0.001	0.077
**Potassium (mmol/L)**	4.4 ± 0.7	4.4 ± 0.7	0.527	0.009	4.4 ± 0.7	4.4 +/- 0.7	0.474	0.014
**Sodium (mmol/L)**	136.6 ± 4.1	136.8 ± 3.9	<0.001	0.060	136.7 ± 4.0	136.8 +/- 3.9	0.034	0.042
**Calcium (mg/dL)**	8.7 ± 0.8	8.8 ± 0.9	<0.001	0.123	8.7 ± 0.9	8.8 +/- 0.9	<0.001	0.118
**Phosphate (mg/dL)**	4.6 ± 1.7	4.7 ± 1.8	0.154	0.022	4.7 ± 1.8	4.7 +/- 1.8	0.144	0.030
**Cholesterol (mg/dL)**	147.5 ± 54.9	152.6 ± 55.4	<0.001	0.094	150.0 ± 55.3	152.6 +/- 55.4	0.050	0.047
**HDL-C (mg/dL)**	42.0 ± 16.9	41.8 ± 17.2	0.509	0.012	42.8 ± 17.7	41.8 +/- 17.2	0.026	0.053
**LDL-C (mg/dL)**	79.0 ± 42.2	81.4 ± 44.0	0.002	0.055	80.9 ±44.7	81.4 +/- 44.0	0.663	0.010
**PTH (pg/mL)**	324.4 ± 347.3	341.6 ± 360.6	0.015	0.049	367.4 ± 391.8	341.6 +/- 360.6	0.011	0.069
**Clinical Measurements**								
**BMI (kg/m^2)**	29.2 ± 7.2	30.1 ± 7.9	<0.001	0.117	29.1 ± 7.4	30.1 +/- 7.9	<0.001	0.130
**LVEF (%)**	52.1 ± 16.2	56.6 ± 13.6	<0.001	0.296	51.6 ± 15.5	56.6 +/- 13.6	<0.001	0.339

Table S5: Survival analysis. Number of patients with each MACE outcome, Hazard ratio and 95% confidence interval for troponin I at a threshold of 50 ng/L.

Outcome	Troponin I							
	<50 ng/L		≥50 ng/L					
	Cohort N=	Outcome N=	Cohort N=	Outcome N=	HR	95% CI	Log-Rank Test χ^2	P-Value
**MACE**	5,739	3,468	3,543	2,410	1.33	1.26 - 1.40	115.04	<0.0001
**IHD**	12,315	4,059	8,809	3,104	1.23	1.18 - 1.29	77.59	<0.0001
**Angina**	27,699	1,495	27,278	1,591	1.22	1.14 - 1.31	30.41	<0.0001
**Acute MI**	23,881	3,843	19,755	3,704	1.33	1.27 - 1.39	151.22	<0.0001
**Heart Failure**	13,432	3,742	11,399	3,249	1.18	1.13 - 1.24	49.25	<0.0001
**Atrial fibrillation and flutter**	21,507	3,214	20,156	3,271	1.22	1.16 - 1.28	62.49	<0.0001
**Cerebral Infarction**	26,669	2,041	26,447	1,915	1.07	1.00 - 1.13	3.97	0.06
**Deceased**	30,766	12,834	30,389	14,933	1.32	1.29 - 1.35	543.43	<0.0001

Table S6: Survival analysis. Number of patients with each MACE outcome, Hazard ratio and 95% confidence interval for BNP at a threshold of 100 pg/mL.

Outcome	BNP							
	<100 pg/mL		≥100 pg/mL					
	Cohort N=	Outcome N=	Cohort N=	Outcome N=	HR	95% CI	Log-Rank Test χ^2	P-Value
**MACE**	1,079	592	749	458	1.28	1.13 - 1.44	15.36	<0.0001
**IHD**	2,173	696	1,906	675	1.21	1.09 - 1.35	12.89	<0.0001
**Angina**	4,666	294	4,680	251	1	0.76 - 1.06	1.71	0.191
**Acute MI**	3,887	636	3,623	668	1.20	1.08 - 1.34	11.18	0.001
**Heart Failure**	2,261	622	1,678	577	1.43	1.28 - 1.60	38.49	<0.0001
**Atrial fibrillation and flutter**	4,020	536	3,685	557	1.20	1.06 - 1.34	8.170	0.004
**Cerebral Infarction**	4,399	353	4,460	374	1.12	0.97 - 1.30	2.44	0.119
**Deceased**	5,180	1,725	5,180	2,094	1.28	1.20 - 1.37	58.61	<0.0001
